# Agent-patient similarity affects sentence structure in language production: evidence from subject omissions in Mandarin

**DOI:** 10.3389/fpsyg.2014.01015

**Published:** 2014-09-16

**Authors:** Yaling Hsiao, Yannan Gao, Maryellen C. MacDonald

**Affiliations:** Department of Psychology, University of Wisconsin–MadisonMadison, WI, USA

**Keywords:** language production, sentence production, subject omission, grammatical encoding, interference

## Abstract

Interference effects from semantically similar items are well-known in studies of single word production, where the presence of semantically similar distractor words slows picture naming. This article examines the consequences of this interference in sentence production and tests the hypothesis that in situations of high similarity-based interference, producers are more likely to omit one of the interfering elements than when there is low semantic similarity and thus low interference. This work investigated language production in Mandarin, which allows subject noun phrases to be omitted in discourse contexts in which the subject entity has been previously mentioned in the discourse. We hypothesize that Mandarin speakers omit the subject more often when the subject and the object entities are conceptually similar. A corpus analysis of simple transitive sentences found higher rates of subject omission when both the subject and object were animate (potentially yielding similarity-based interference) than when the subject was animate and object was inanimate. A second study manipulated subject-object animacy in a picture description task and replicated this result: participants omitted the animate subject more often when the object was also animate than when it was inanimate. These results suggest that similarity-based interference affects sentence forms, particularly when the agent of the action is mentioned in the sentence. Alternatives and mechanisms for this effect are discussed.

## INTRODUCTION

An important tool to understand the mapping from conceptual to lexical representations during language production is the picture-word interference paradigm, in which speakers name a picture and attempt to ignore a word printed on it. Picture naming in this situation is influenced by the relationship between the target picture and the distractor word. One classic result is that naming of a target picture (e.g., *cat*) is slower when the distractor word is of the same semantic category (e.g., *dog*) than when the word is semantically unrelated (e.g., *clock*; e.g., Rosinski et al., 1975; Glaser and Düngelhoff, 1984, and many studies since). This result is interpreted to support the claim that lexical selection (settling on the word *cat* to name the picture) is a competitive process and is subject to interference from other activated words, in this case the highly semantically similar distractor word *dog*, making it harder to settle on the correct item for the utterance plan. This effect is reminiscent of behavior in the Stroop task (Stroop, 1935), where people are slower to name a color patch in the presence of a distractor color word (again, an element from the same semantic category). The effect may also be related to phonological interference among items in an utterance plan, in which partial phonological overlap among words leads to longer initiation latencies and higher levels of production errors. Although the picture-word paradigm yields a mix of interference and facilitation effects from phonological similarity, depending on timing and other factors (e.g., Schriefers et al., 1990; Meyer and Schriefers, 1991), phonological overlap in phrase and sentence production yields longer latencies and more errors (Wilshire, 1998; Acheson and MacDonald, 2009; Janssen and Caramazza, 2009; Jaeger et al., 2012).

The sum of these results suggests that while planning an utterance, certain properties of words may interfere with one another in a variety of ways. There is still much to be learned about the nature of this interference, but this article addresses instead a *consequence* of interference in language production. In many of the studies noted above, the participants are constrained in the order in which they utter the words, or, in the case of picture-word interference tasks, have only one word to utter. In more unconstrained sentence production, however, producers often have a choice of word orders and sentence structures to convey an intended message. We investigate whether same-category interference, such as between *cat* and *dog*, also affects the sentence structures and word order that is developed during grammatical encoding. That is, we ask whether speakers’ and writers’ implicit choices of sentence structure are different in situations conveying a message with two same-category competitors (e.g., *cat, dog*) vs. situations in which the message does not require two same-category lexical items. Specifically, we speculate that the message is realized by the producer in a way that minimizes competition between the two semantically similar items. As a first step, our investigations compare messages with two animate sentence participants, so that the producer needs to convey that a human is acting on another human, to those messages in which a human is acting on an inanimate object. For now, we will set aside the issue of whether any interference between the animate entities exists at a conceptual or lexical level or both (see Damian and Bowers, 2003, for discussion) and will simply refer to any interference of this sort as similarity-based interference.

## PRODUCTION CHOICES AND PRODUCTION DIFFICULTY

Almost any concept or message can be conveyed in a number of different ways—different sentence structures, word orders, and lexical items. MacDonald (2013a) argued that during the stages of utterance planning that precede articulation in language production, producers tend to settle on utterance forms that minimize the difficulty of utterance planning. She argued that these effort minimization biases were emergent from non-linguistic action and motor planning, where easier (more practiced, simpler, recently used, etc.) motor plans capture internal attentional resources over more complex plans, and they are therefore more likely to be implemented than the more complex alternatives. In incremental language production planning, in which elements of the utterance plan are developed and held in memory before the plan is executed, the presence of several semantically similar elements in the plan reduces the distinctiveness of these elements in memory (Acheson and MacDonald, 2009), thereby increasing the difficulty of developing and maintaining the ordered elements in the plan. One difficulty-minimization utterance planning bias that MacDonald identified was Reduce Interference, that producers tend to develop utterance plans that minimize similarity-based interference. Here we consider how similarity-based interference could affect the accessibility (readiness for articulation, Bock and Warren, 1985) of the interfering elements, and the consequences of variation in accessibility for choices of utterance form during language production.

A number of studies have shown clear effects of lexical accessibility on sentence structure and word order in production, so that more conceptually salient (accessible) nouns tend to be placed earlier in the utterance or at a syntactically more prominent position (e.g., Bock and Warren, 1985), such as the grammatical subject. For example, animate nouns, which are thought to be more salient and recalled more rapidly from long-term memory, are more likely than inanimate nouns to be uttered early and assume the surface subject position even when they are not agents of the event, resulting in the production of passive sentences like “*The boy was hit by the ball*,” rather than the active form “*The ball hit the boy*” (Bock et al., 1992). Structural relations of nouns in the sentence, in this case in English, are affected by the conceptual roles associated with the animacy of the referents of the nouns. Similarly, data from languages that allow flexible word order, such as Japanese, show that sentences in the object-subject-verb (OSV) word order with animate subjects tend to be recalled as subject-verb-object (SVO), associating animate nouns to the more prominent subject grammatical role, and to an earlier sentence position (Tanaka et al., 2005). These and similar results concerning the effects of accessibility on sentence form are relevant to effects of similarity-based interference, because if similarity-based interference can affect the accessibility of words to be placed in the utterance plan, then it is plausible that these variations in accessibility could affect sentence form.

A second piece of evidence that makes it plausible that similarity-based interference could affect word order is that similarity-based interference affects utterance planning difficulty. Semantic or phonological similarity increases the rate of serial ordering errors in production: Dell and Reich (1981) studied the rate of word exchange errors, such as when the intended message *I wrote a letter to my mother*, is realized as *I wrote a mother to my letter*. They found that the exchanged words (e.g., *letter, mother*) have more phonological similarity than would be expected by chance, suggesting that the phonological similarity increases the chances of ordering errors during language production planning. Semantically related items in the utterance plan also yield longer initiation latencies, longer utterance durations and overall higher error rates, compared to conditions without semantic similarity. Acheson and MacDonald (2009) relate these and other similarity effects to contextual distinctiveness in serial ordering, in which nearby items in a memory representation (including an utterance plan during language production planning) tend to have more similar contextual representations than those farther apart, regardless of whether the contextual representation is external (e.g., list position in a recall task) or internal (e.g., distributional properties of syllable position in individual words: vowels and consonants are less likely to substitute each other in speech errors) to the items. In the short-term memory literature, similar constraints also apply: with short interval between presentation and recall, items interfere with one another in memory when sharing similarity in sound, meaning, location, or other dimensions (Anderson, 1983). Utterance planning has similar short-term memory demands (Acheson and MacDonald, 2009; MacDonald, 2013a), and so these same distinctiveness effects would be expected to influence serial ordering in language production, such that two similar (less distinct) items would be more likely to be exchanged or subject to error than two more distinct items.

Together these findings suggest that (a) similarity between entities in an utterance plan affects the difficulty of the planning of that utterance and the likelihood of errors, (b) similarity affects the accessibility of entities in the utterance plan, and (c) the accessibility of items influences sentence form and word order. Gennari et al. (2012) investigated the effect of similarity on sentence form using picture description tasks in three languages: English, Spanish, and Serbian. They studied active vs. passive relative clause production, as in *The baby that the woman is holding* vs. *The baby that’s being held by the woman*. Unlike simple active and passive sentences, which have different noun orders, relative clauses in these three languages fix the position of the modified noun (i.e., head noun, *baby* in this example) in the clause-initial position and therefore allow better comparison between the active and passive forms. Thus in the active form *the baby that the woman is holding*, the head noun *baby* and embedded subject *woman* are near each other and are both in prominent grammatical roles (*the baby*: main clause subject, *the woman*: relative clause subject). In the passive, however, the agent of the holding action, *woman*, is produced in the by-phrase, which is optional. Gennari et al. (2012) found that in picture descriptions in all three languages, the rate of passives was higher when both entities in the picture were animate (e.g., a woman holding a baby) than when an animate entity was acting on an inanimate entity (e.g., a woman holding a vase). Moreover, within the set of passive utterances, the rate of agentless passives, omitting the by-phrase, was higher for the animate patients (*The baby that’s being held*) than for the inanimate ones (*The vase that’s being held*). These results, which were replicated in English by Montag and MacDonald (2014), suggest that interference between the conceptually similar items *woman* and *baby* reduces the accessibility of *woman* and thereby promotes the passive form, where the agent *woman* is either demoted to a more minor part of the sentence (the by-phrase) or omitted entirely in the agentless passive.

To test that this effect stemmed from agent-patient similarity and was not simply an effect of animacy of a noun, Gennari et al. (2012) collected similarity ratings for the entities in their pictures. They found that in both Spanish and English (the two languages in which there were enough passives to conduct the analyses), the more similar the two entities to be described were, the more agentless passives were produced. A follow-up experiment using new pictures with only animate event participants confirmed this pattern: in both Spanish and English, participants produced more agentless passives (e.g., *The builder who was slapped*) when the agent was semantically similar (a miner) than when the agent was dissimilar (an astronaut). These results show how structure choice can emerge not simply from properties of a single noun, such as the head noun *builder* but also via the interaction between two event participants. When these entities are highly similar and thus create similarity-based interference, producers are more likely to omit mention of one of them from the utterance.

These studies shed light on potential underlying causes stemming from production constraints for the utterance forms that constitute distributional regularities in a person’s language experience (MacDonald, 2013a). Most of the evidence for the biases mentioned above, however, comes from complex constructions, such as relative clauses. One concern is that a multitude of complex interactions among production constraints and task demands can be at work to create the patterns that Gennari et al. (2012) observed in relative clause production. Therefore, instead of using complex structures like relative clauses, we chose to examine simple sentences in Mandarin Chinese, a language that allows noun omission in certain discourse contexts. Typically the omitted element is thought to be a pronoun, because the discourse environment in which omission is possible is also the environment (prior mention) in which it is felicitous to use a pronoun. The omission phenomenon is variously described as pro-drop (i.e., that a pronoun is dropped), pronoun elision (i.e., omission), and null subject and null object, referring to an omission of the grammatical subject or object, respectively. Some languages, such as Spanish, permit omission of only the subject, while others, such as Mandarin and Japanese, permit omission of subjects, objects, and some other grammatical positions. Although omission phenomena have received a number of linguistic treatments, syntacticians commonly view sentences with omitted elements to have a different syntactic structure than the sentences in which the pronoun is present, although analyses may differ by languages (e.g., Biberauer et al., 2010; Camacho, 2013)

As our focus here will be on omitted subjects, we will refer to *omitted* or *null subjects*, even though Mandarin also allows omission of other grammatical positions. For example, in a scenario where two Mandarin speakers have been talking about a movie, one person can ask the other the question “Did you watch the movie?” in four different formats: (a) “You watched the movie?,” in which both you and movie are overtly mentioned, (b) “You watched __?,” in which movie is omitted from the utterance, (a null object construction), (c) “___ watched the movie?,” a null subject construction, or (d) “___ watched ___?” in which both the subject and object are omitted from the utterance. All four of these alternatives are grammatical in Mandarin, and the clarity of the message is not compromised as long as the context provides clear clue to what the omitted elements are, much as the message is clear in the English, “Want to go to a movie?,” in which the pronoun *you* is omitted. Unlike many other pro-drop languages, Mandarin lacks a rich morpho-syntactic system that redundantly encodes the pronominal information with verbal inflections and other agreement systems (no number, gender, and tense agreement, no case marking). Therefore, Mandarin pro-drop may lend us a clearer lens in uncovering the production mechanisms behind null subjects and other omissions, perhaps more purely based on the lexical retrieval difficulty among the competing nouns.

In two studies reported below, we investigated the role of similarity-based interference on producers’ use of null subject constructions in Mandarin. If the Gennari et al. (2012) relative clause production phenomena (i.e., agent omission in relative clause production in English and Spanish) generalize to a very different language and sentence structure, then producers should produce more null subject structures when the subject and object are similar than when they are dissimilar. We investigated this prediction in an analysis of a written corpus in Study 1 and in a spoken picture description task in Study 2, using animacy of the subject and object nouns as a proxy.

## STUDY 1 – CORPUS ANALYSIS

The corpus analysis presented here is an extension of one originally conducted by Hsiao and MacDonald (2013). Their original analysis focused on main and relative clause usage in Mandarin, with the goal of creating a training set for a computational model that closely matched Mandarin speakers’ experience relevant to Mandarin relative clause comprehension. Among other sentence types, Hsiao and MacDonald extracted all simple (one clause) sentences with overt or null subject noun phrases from the parsed Chinese Treebank 7.0 (Xue et al., 2010). There were 4035 simple transitive sentences with overt direct object phrases, of which 2445 (61%) contained overt subjects and 1590 (39%) contained null subjects. These 4035 sentences formed the basis for our analyses here.

Hsiao and MacDonald (2013) hand-coded the animacy of all overt noun phrases in these sentences, but they did not code the animacy of the referent of the (omitted) subject nouns in the null subject sentences, that is, the animacy of the entity being discussed in the broader discourse context. In order to investigate whether null subject sentences are more frequent when the subject and object are conceptually similar than when they are less similar, we used the surrounding sentence context to code the animacy of the intended referents for the omitted subjects.

The 1590 null subject sentences were coded for the animacy of their omitted subjects. Two native Mandarin speakers who were blind to the hypotheses coded animacy of the null subject via the material in the verb phrase. For example, when the sentence read “___ gave a thank-you speech,” the verb denotes an action that could only be completed by a human. Therefore, the omitted subject NP was coded as animate. For sentences like “___ exceeds the percentage last year,” the omitted subject refers to some numerical value, which was coded as inanimate. Sentences for which the verb phrase did not clearly convey subject animacy, such as “___ created uproar,” were coded as ambiguous. The overall inter-rater reliability was 85%. All items with a disagreement among coders were excluded from further analyses.

### RESULTS

**FIGURE 1:**
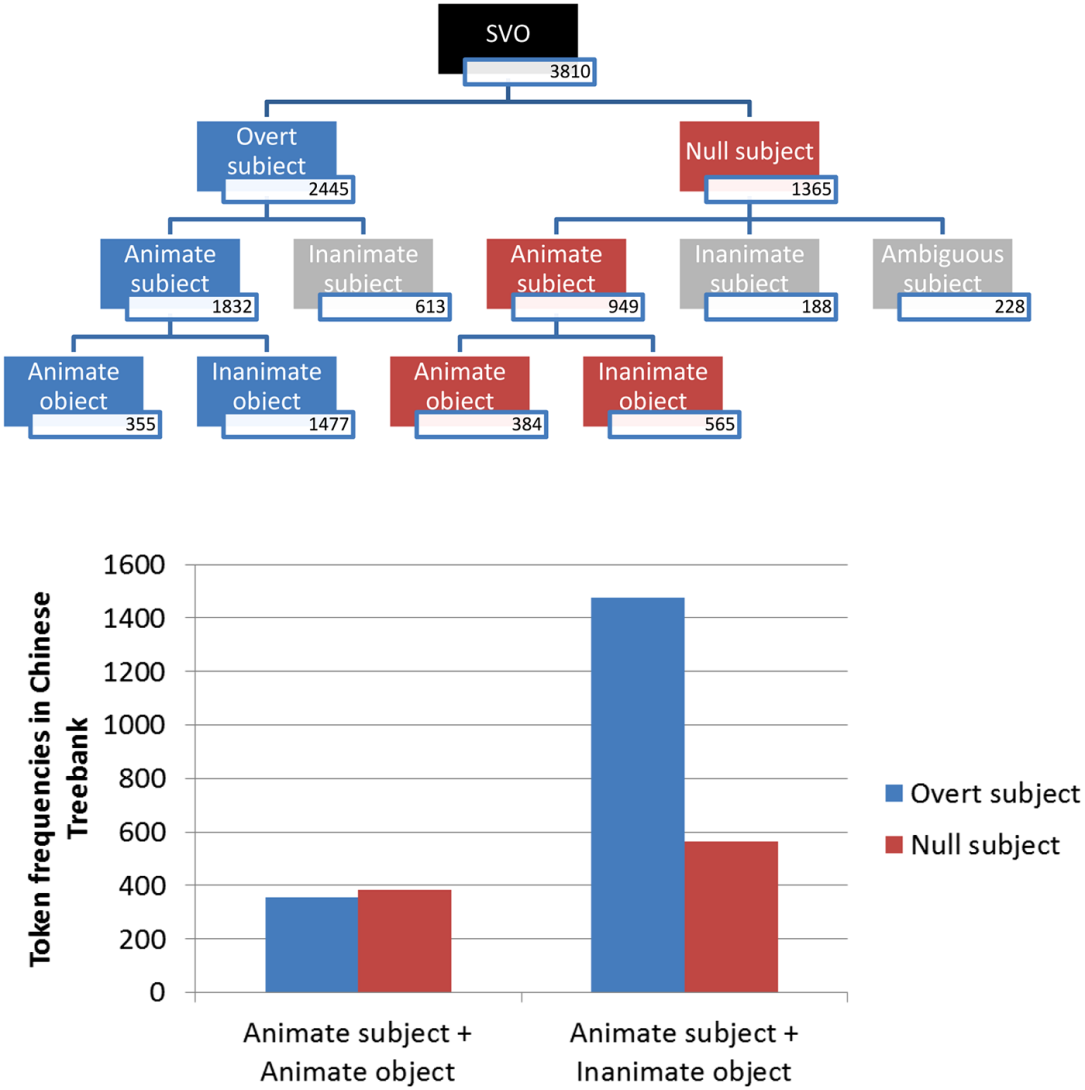
**The flow chart presents counts of types of sentences extracted from the Penn Chinese Treebank, excluding coding disagreements.** SVO, subject verb object, i.e., transitive sentences in the canonical word order for Mandarin. The grayed boxes are types of sentences excluded from further consideration in the current study. The lower graph displays the token frequencies of overt subject simple sentences and null subject sentences grouped by subject and object animacy.

The coding results are summarized in the flow chart in **Figure 1**. Among a total of 1365 null subject sentences after excluding coder disagreements, 949 sentences were coded by both raters as having animate referents for the null subjects, 188 were coded as having inanimate subjects, and 228 were agreed to be ambiguous, meaning that subject animacy could not be determined from the sentence context. Since animacy could not be established for the ambiguous items, they were excluded. We also excluded sentences with inanimate subjects, because there were too few observations in each cell when these items were partitioned into groups with animate vs. inanimate direct objects.

Among the 949 sentences with animate subject referents, 384 items had animate objects, and 565 were with inanimate objects. The bar graph in **Figure 1** compares these values to the patterns of overt subject usage that Hsiao and MacDonald (2013) found. Overt animate nouns, on the other hand, contained 355 sentences with animate objects, and 1477 with inanimate objects, These data show that there was a strong association between subject omission and the animacy of the direct object: when both the subject and the object were animate, the frequency of null subject sentences was higher than that of overt subject sentences; whereas when the subject was animate and the object was inanimate, the majority of them were overt subject sentences, [χ^2^(1, *N* = 2781) = 142, *p* <0.05].

These results are consistent with the hypothesis that in conditions of similarity-based interference, speakers produce more null subject sentences. We also considered a second possibility, that similarity-based interference could affect the use of overt pronouns vs. full noun phrases, as some previous research has suggested that pronoun use varies as a function of whether the animacy of subjects and objects matches or not. Fukumura and van Gompel (2011) and Fukumura et al. (2011) found that in sentence completion tasks where the subject and object NPs were of the same animacy, participants referred to either one of them (depending on the manipulation: half of the time the subject NP and the other half the object NP) with pronouns less frequently than when both NPs were of different animacy. The finding suggests that similarity in meaning between the two nouns makes the referent’s representation less accessible. However, in our study, the pronoun/full noun phrase contrast could not be investigated, because subject pronouns were too rare—the vast majority of sentences contained overt full noun phrases or null subjects, and subject pronouns comprised only about 3% of the extracted sentences. The low percentage of pronoun use may be attributed to the formal nature of written texts in Mandarin Chinese. Mandarin overt pronoun use varies with the social distance between the speaker and the interlocutor, and even the third party being referred to. The farther the social distance between the producer and the referent, the less likely a pronoun will be used (rather, role names are used for higher-ups, e.g., addressing your college professor as “Professor Wang” instead of “you”). This explains the rarity of pronoun use in the current corpus, which is composed of articles and transcripts from newspapers or news broadcasting normally written with formal language (Wang, 1987; Wang, 2007).

### DISCUSSION

The corpus results suggest that when the agent and patient are of similar and salient conceptual representations (animate entities), people producing a simple transitive sentence are more likely to omit the subject (agent). This pattern, as seen in unconstrained natural speech transcripts and texts outside of the laboratory, is a valuable piece of evidence for the relationship between similarity-based interference and subject omission in production. However, as with any unconstrained language sample, we cannot be sure whether other factors instead of or in addition to agent-patient similarity affected subject omission. For example, the sentences with animate direct objects may have tended to occur in different kinds of discourse contexts than those with inanimate objects. The use of null subjects is dependent on the referent being previously established (given) in the discourse, and it is possible that higher rates of null subjects in the animate direct object sentences may have been due to those sentences appearing in discourses in which the agent of the action had been more firmly established in the discourse compared to the sentences with inanimate direct objects. To address this concern, in the next experiment, we conducted a picture description task that controlled the discourse contexts to be equally plausible and appropriate for subject omission in all conditions and manipulated the animacy of patients/themes in the event while keeping the agents animate. If similarity-based interference affects the rate of subject omission in production, then we should find a similar pattern to the one in the corpus analysis: more subject omission when both the agents and the patients of the action are animate.

## STUDY 2 – SENTENCE PRODUCTION TASK

### PARTICIPANTS

A total of 26 native Mandarin speakers were recruited from an Introductory Psychology class at the University of Wisconsin-Madison. All participants reported that they had been born or educated in China or Taiwan and spoke Mandarin Chinese as their dominant language. The majority of them were freshmen and sophomores who had spent less than 2 years in the United States. Participants received extra credit in the course for participation in the study.

### MATERIALS

All pictures for the experiment were created using the online comic design website Pixton^1^. Twenty experimental picture triples were created. One member of the triple was an *introductory* picture, depicting a single standing human character with neutral facial expression. This picture introduced the agent of a subsequent action, creating a discourse context in which it would be felicitous to use either an overt pronoun or a null subject construction when referring to this character. The other two pictures were *action* pictures and showed the character acting on another entity. In one version, the entity being acted on was animate (another human), and in the other, it was inanimate.

**FIGURE 2:**
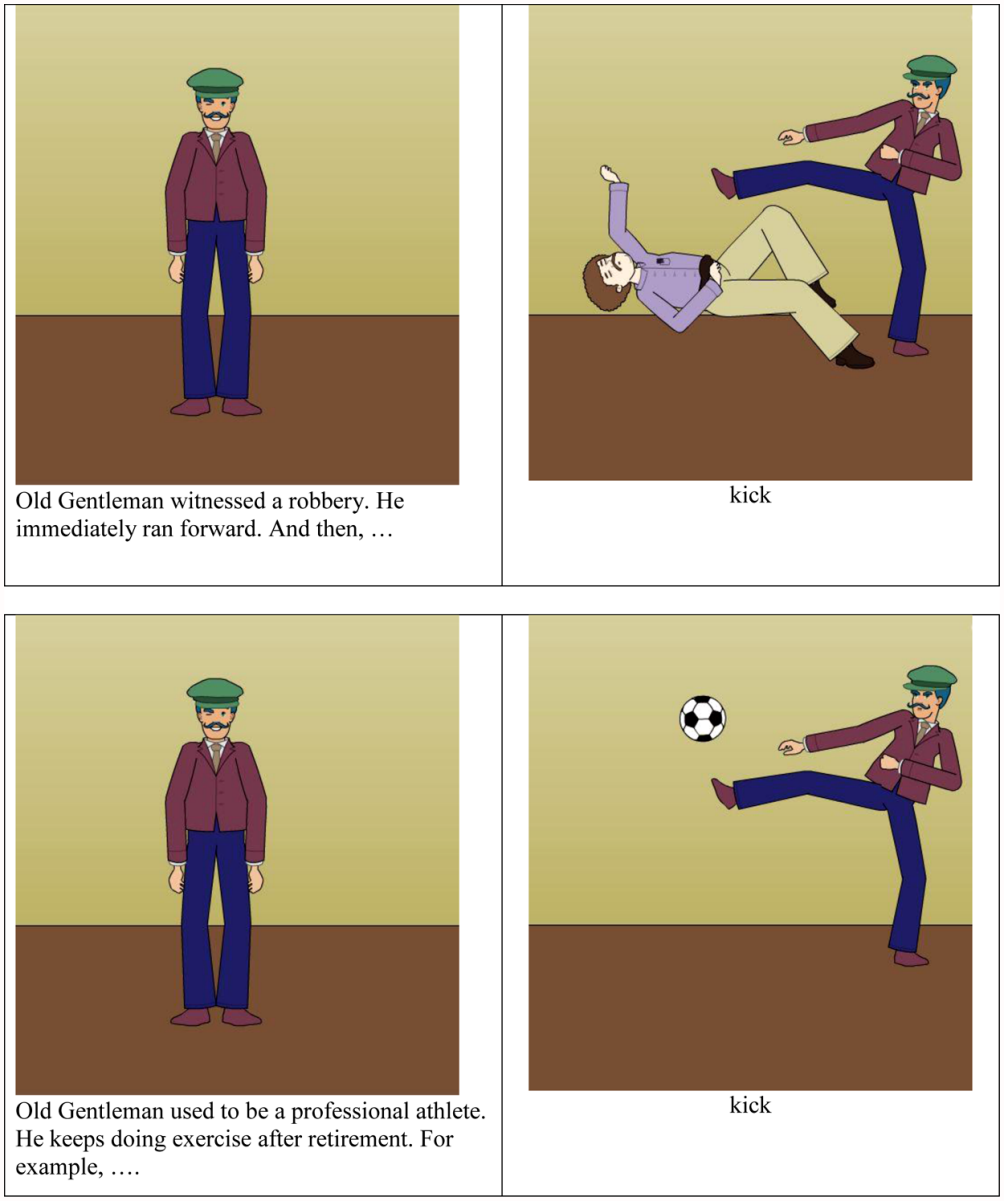
**Example experimental items.** Each trial consisted of a pair of pictures, with an introductory picture and text on the left and action picture on the right. The object of the action was either animate (upper pair) or inanimate (below), with the verb in the text below the action picture. Participants saw only one picture pair.

The introductory picture was paired with one of the action pictures in each trial, with the introductory picture arranged to the left of the action picture. An example is shown in **Figure 2**. The two pictures were presented together in order to create a sense of continuous story flow and thus a better discourse environment for subject omission. Two or three sentences were written under the introductory picture, providing background information about the character (e.g., occupation, disposition, habits) and establishing the character as given in the discourse. The character’s label was used as the grammatical subject of the first sentence (e.g., *Old Gentleman* for the examples in **Figure 2**) and a pronoun referring to the pictured character as the grammatical subject was used for subsequent sentences (e.g., *he*). In addition to introducing the character into the discourse, these introductory sentences also served to establish the plausibility of the event conveyed in the action picture. Because it was difficult to provide a single plausible discourse context for both an event involving an animate patient and one involving an inanimate object, the contexts differed for the two conditions where necessary to create a plausible sequence of events.

The action picture on the right appeared with a single word referring to an action, in order to encourage all participants to be consistent in their verb use when describing the action picture. For test trials, the action picture always depicted a transitive action performed by the human character introduced in the picture on the left. The human character exerted the action on an animate patient or an inanimate theme in the picture on the right. The two versions of the action pictures were controlled to have the same background color and the same human character, which was made to have roughly the same action and position in the two action pictures. Thus the only difference between animate and inanimate action picture was the animacy of the object of the action. The verb used to describe the action was selected to be appropriate for both an inanimate and animate object. Two lists were created to counterbalance the assignment of animate or inanimate action pictures across participants, each of whom saw 10 animate and 10 inanimate objects in the experimental action pictures, and no participant saw both versions of the action pictures for a given item.

Thirty filler picture pairs were created. These were similar in form to the experimental items except that there was only one action picture matched with an introductory picture, and some of the action pictures depicted intransitive actions with no direct object. On some filler trials, the word under the action picture was a noun rather than a verb.

### PLAUSIBILITY NORMING

In order to ensure that the pairs of introductory and action pictures were equally plausible in the animate and inanimate object conditions, we conducted a rating study with a separate group of 48 native Mandarin-speaking participants, all of whom were from mainland China. The survey took 7–10 min to complete. Participants volunteered their time and were not compensated for participation.

The rating task had 20 test trials and 20 filler trials, each with introductory and action pictures with associated text, except that the single word underneath the action pictures that appeared in the main experiment was not presented in the rating study. The filler trials were 20 of the filler picture pairs from the main experiment, except that a portion of them had their text modified to be less plausible. This change was designed to create some variability in the range of events and to provide a manipulation check to determine whether raters were reading the text carefully and assessing plausibility in each trial.

Two lists were created, counterbalancing assignment of inanimate or animate action picture across subjects, so that each participant saw 10 animate and 10 inanimate objects in the experimental action pictures, and no participant saw both versions of the action pictures for a given item. The participants were asked to indicate the plausibility of the event in the action picture given the context sentences for the introductory picture, using an on-screen sliding scale of scores 1–7, with 1 referring to extremely implausible and 7 to very plausible. The study was hosted through the online surveying service Qualtrics^2^. On each trial, participants saw a picture, read the associated text, and used the mouse to adjust a slidebar on screen to correspond to their plausibility rating. Participants proceeded through the items at their own pace.

Statistical analyses of the plausibility data were conducted with mixed effects models with maximum random effects of participants and items, as suggested in Barr et al. (2013). The plausibility of the filler trials were rated significantly lower than experimental trials, with an average of 3.37 for fillers and 5.21 for experimental trials (β = 1.84, SE = 0.26, *t* = 7.20, *p*<0.001). The fact that the average ratings of fillers and experimental items fell on the opposite sides of the neutral rating of 4 confirmed the success of our design to involve overall plausible events for the experimental items and for a portion of the fillers to be less plausible. These results also suggest that participants were reading carefully when rating the picture pairs. We further analyzed the ratings within experimental trials and found no difference between the ratings for the animate condition and those of the inanimate condition, with the former having an average of 5.03 and the latter 5.39 (β = 0.36, SE = 0.22, *t* = 1.6, *p* = 0.11). Thus even though inanimate direct objects are more common in the world (e.g., as in the corpus analysis in Study 1, in which inanimate objects were more common than animate ones at a rate of about 2:1), the null result here suggests that the discourse contexts we designed made the inanimate and animate conditions similarly plausible.

### PROCEDURE

E-prime 2.0.10 was used to create experimental scripts for the main production experiment. Participants were assigned to one of the two lists, each containing 20 test trials and 30 filler trials. These trials were interleaved so that no more than three test trials appeared in a row.

In each trial, participants were asked to read the context sentences under the introductory picture aloud and then continue with a description of the character’s action depicted in the action picture, using any sentences regardless of structure and length, as long as the response contained the verb shown below the action picture. Participants were encouraged to describe the action picture soon after finishing reading the context sentences aloud, without pausing to consider elaborate continuations. Before the experiment started, participants practiced with two sample trials. Participants’ responses were recorded digitally through a microphone.

### RESULTS AND DISCUSSION

Participants’ responses were transcribed and coded by a native Mandarin speaker. Utterances in which the agent was not the grammatical subject of the main clause (e.g., appearing in a conjunction clause or a passive sentence) were excluded from analyses. A total of 11% of responses were excluded. Responses were coded as null subject utterances when the grammatical subject position was empty and coded as overt subject utterances when a word occupied the subject position. All of the overt responses were pronouns; there were no responses that repeated the full NPs (i.e., character descriptions such as *Old Gentleman*) or used new descriptions such as *the old man*. The lack of repeated NP or full NP descriptions suggests that the introductory picture did establish the agent as given, allowing an overt pronoun or null subject continuation. The common use of overt pronouns in the spoken descriptions, in contrast to their rarity in the corpus, may have stemmed from the same social-discourse factor mentioned before: the speech modality here, and possibly the topics mentioned in the context sentences, are less formal than in the primarily written texts extracted from the corpus in Study 1.

**FIGURE 3:**
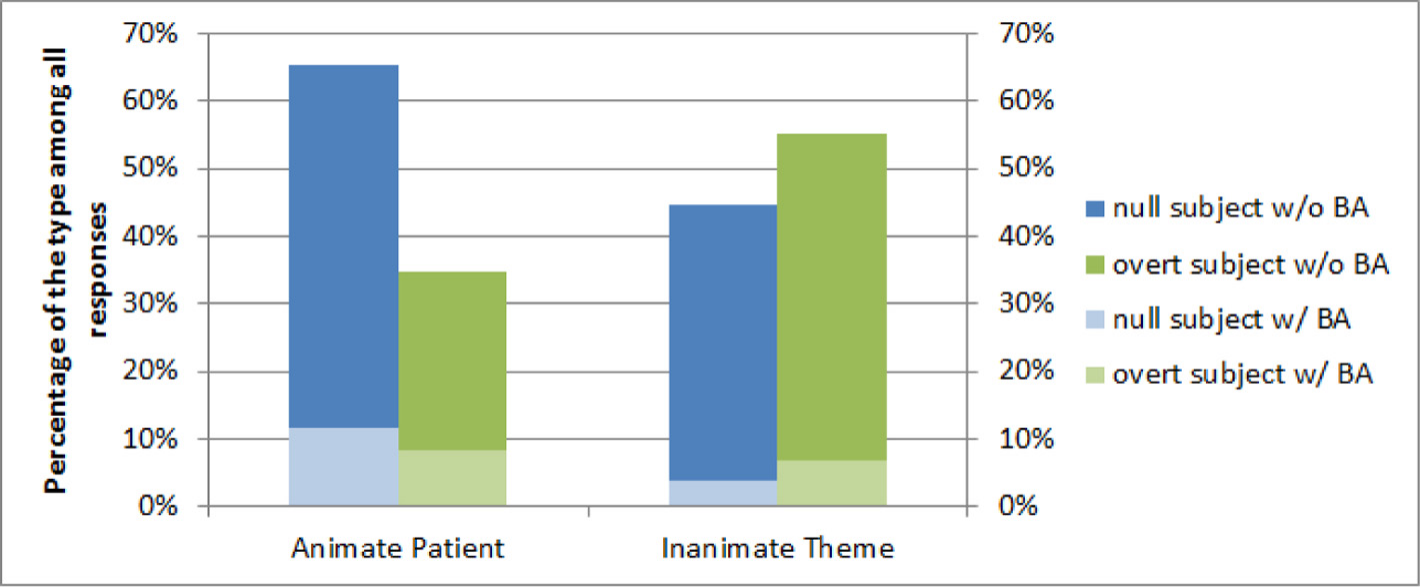
**The rate of overt subject (green bars) vs. null subject (blue bars) responses in the picture description task.** The blue and green bars in each animacy condition add up to 100%. The lighter part of each bar indicates productions using the disposal constructions, and the darker portion shows productions with the standard SVO word order.

Rates of subject omission in animate and inanimate object conditions are shown in **Figure 3**. Statistical analyses of participants’ utterances employed mixed effects models with maximum random effects of participants and items (Barr et al., 2013). Comparing the rates of subject omission between the two conditions, we found that when the human character acted upon an animate patient, speakers omitted the subject NP 65% of the time, which was a reliably higher omission rate than when the human agent acted upon an inanimate object, with 44% omissions, (β = -0.21, SE = 0.10, *t* = -2.12, *p* = 0.04). The animacy effect remained significant after adding the plausibility ratings from the norming study as an additional factor to the model, and plausibility itself did not account for significant amount of variance in the responses (*t* = 0.01). Given these animacy effects, a logical next step would be to identify whether finer-grained level of similarity beyond animacy should also show an effect on subject omission, as in the all-animate condition of Gennari et al. (2012). For example, pictures with two more similar human characters (same gender, similar age, occupation) could yield more null subjects than for pictures with more dissimilar human characters. We leave this to future research.

Some of the participants’ responses employed the Mandarin disposal construction, also called BA construction (see **Figure 3**), which is a common form in describing Mandarin transitive events and typically expresses how an entity is handled, manipulated or dealt with (Li and Thompson, 1981). This construction was not included in the corpus analysis, which focused on simple sentences with SVO word order. In the production study, 85% of utterances were in this SVO word order and 15% were in the disposal construction, which has an SOV word order, with a light verb, such as *ba* or *jiang*, inserted between the subject and the object, as in *he* ***ba*** *robber kicked*, in the overt subject variant, or ***ba*** *robber kicked* in the null subject variant. The disposal construction is interesting from a production standpoint because it affords the producer an alternate word order, but the factors that promote use of this construction are beyond the scope of the current paper. Accordingly, our analyses focused simply on whether use of the disposal construction interacted with subject omission in some way. As **Figure 3** shows, there was a numerically higher percentage of disposal construction sentences in the animate condition (20%) than in the inanimate condition (11%), but this difference did not reach statistical significance (*p* = 0.1) after including maximal random effects of subject and item. To test whether the use of the disposal construction might be related to subject omission, we added the percentage of null subjects as a factor in the model predicting disposal construction use, but the result was again not reliable (*p* = 0.9). These results suggest that while the factors that promote production of the disposal vs. simple transitive construction are interesting and merit further study, the rate of subject omission does not appear to be tied to use of the disposal construction in this study.

## GENERAL DISCUSSION

The current study explored the effect of similarity-based interference in sentence production, using the presence of two human sentence participants as a condition of high similarity and a low similarity condition in which an animate entity acted on an inanimate one. The findings from both corpus analyses and a picture description experiment suggest that when Mandarin speakers are faced with developing an utterance plan containing two conceptually similar entities that may interfere with one another, they are more likely to omit one of the interfering elements than in the low similarity conditions.

The Mandarin null subject results here are similar to Gennari et al.’s (2012) results with relative clauses in English and Spanish, with higher rates of agentless passives (omission of the by-phrase) with a similar patient than a dissimilar one. Putting these results together with the ones in the current studies, there are consistent effects of agent-patient similarity on agent omissions across three quite different languages—English, Spanish, and Mandarin, across two sentence types—simple transitive sentences and relative clauses, and across paradigms—corpus analyses and picture descriptions. Together these results point to effects of similarity-based interference on utterance form, specifically in choice of sentence structures that allow omission of the agent of the action—the null subject structures in the current studies, the agentless passives in English and Spanish in Gennari et al. (2012) and in Spanish a third agentless “impersonal” construction that Gennari et al. (2012) found is also more common under conditions of similarity-based interference. Thus over several different languages and structures, the unifying theme seems to be increased agent omission when the agent and patient of an action are similar compared to when they are less similar. In the next sections, we consider the evidence and opportunities for future research investigating the possible mechanisms underlying this agent omission effect, its relationship to other phenomena in production, and implications for theories of language production.

### INTERFERENCE, ACCESSIBILITY, AND INCREMENTALITY

There are several potential mechanisms that could link the similarity-based interferences effects in picture-word interference studies and the agent omissions that we’ve observed in sentence production. One possibility is that agent omission is an implicit strategy in language production: faced with interfering elements during utterance planning, speakers strategically choose an utterance form that reduces interference, i.e., choosing a form in which one of the interfering elements is placed some distance (in words) from the other, where the interfering elements are placed in very different syntactic positions (such as grammatical subject and adjunct, as in passives such as *The boy who was pushed by the girl*), or where one element is omitted altogether. On this view, structure choice is a direct (though unconscious) strategy to limit the interference and maintain fluency during production. An alternative view is that the utterance form is simply a consequence of the accessibility of the elements. On this more emergent view of omission, interference between similar elements leads to at least one of these elements being relatively inaccessible during utterance planning, with consequences for utterance form, as in other studies of accessibility in language production. Those studies often aim to increase an element’s accessibility, via priming, question-focusing, repetition, or other manipulations, with the consequence that speakers are able to retrieve highly accessible elements early and thus utter them early in an utterance (Bock and Warren, 1985; Bock, 1986). Interference has the opposite effect, decreasing accessibility, so that these low-accessible elements are delayed or omitted in the utterance. Thus both approaches link interference, accessibility of elements, and utterance form, but they differ in the extent to which they view this sentence-level planning phenomenon as strategic vs. emergent from the accessibility of elements of the utterance plan.

We do not believe that the experiments presented here or elsewhere distinguish these alternatives, and indeed it is not clear that the alternatives are completely incompatible. At issue is really the extent to which sentence planning is or can be under strategic control, which would accommodate strategic use of utterance forms to reduce interference between elements. Sentence form clearly can be under some deliberate strategic control on some occasions, and poets and other writers do consciously choose some sentence forms in some circumstances. It is less clear whether sentence form is always under a degree of strategic control, or whether it is more purely emergent from accessibility considerations at other times. The debate here seems similar to the question of the degree to which incrementality (planning ahead) during language production is under strategic control. Previous research does point to some amount of strategic control in the degree of advance planning (Ferreira and Swets, 2002; Wagner et al., 2010).

The analogy to incrementality here is interesting because the current data also bear on the question of the degree of advance planning during sentence production. By definition, similarity-based interference implies activation of both interfering entities, and therefore it suggests that there is sufficient advance planning to allow both entities to affect the development of the utterance plan. As such, the interference effects here argue against a “radical incrementality” perspective in which the first element (typically the subject) is planned and the sentence structure is adjusted thereafter to fit this encoding (e.g., Kempen and Hoenkamp, 1987; Levelt, 1989; de Smedt, 1996). Indeed, the Mandarin results are striking in this regard because material to be produced downstream (material in the verb phrase) affects whether the first position (the subject) will be uttered or not. Thus the current results are more consistent with a view in which an incremental production system is under some degree of strategic control of the speaker, and in which more advance planning may take place before production begins (Ferreira and Swets, 2002; Allum and Wheeldon, 2007; Wagner et al., 2010). This work is also consistent with results of Christianson and Ferreira (2005), who studied Odawa, a free word order language, using a picture description task that manipulated the agent and patient animacy and the focusing question. When the questions focused on an animate patient (e.g., “What is happening to the girl?” for a picture depicting a girl being pinched by a boy), participants’ answers tended to be passives even though the active object-first structures are appropriate answers, such as object-verb-subject or OSV. This result suggests that speakers would choose an overall less frequent sentence structure (i.e., passives) even though the language allows the dominant active voice structure to appear with many word orders. Their results suggest that structure does not simply emerge from putting the most active element in sentence-initial position.

As researchers pursue these agent omission phenomena and the mechanisms that underlie them, it will be important to connect this work to another literature, the one addressing choice of referential form. That is, here we have been considering choice of sentence form, such as whether producers converge on an active or passive sentence, a full or agentless passive, or an overt or null subject, and most syntactic analyses consider these alternatives different syntactic constructions. However, the choice of a null vs. overt mention of an agent is also a choice of referential form—how producers choose to refer to some entity in the message. Typically studies in that literature investigate the conditions under which producers use (overt) pronouns vs. full noun phrases such as *the boy* or *Mary* (e.g., Arnold, 2010; Fukumura and van Gompel, 2011; Fukumura et al., 2011), but clearly speakers also choose omission to “refer” to entities for some languages, under certain discourse conditions and levels of interference. Indeed, some pronominal reference work describes cost functions for different referential forms (Almor and Nair, 2007). This point raises a related question: if the similar interfering elements (such as *cat-dog* or *old gentleman-robber*) are part of the producer’s message and thus a part of utterance planning, why is it that specifically overt mention is difficult? The answer, or perhaps a re-description, is that overt articulation appears to be especially sensitive to similarity-based interference. That is, perceiving or thinking about related elements (such as a cat and dog or an old man and robber) may not be more difficult than perceiving or thinking about less related ones (and may even be easier, given associative priming between related elements that is commonly found in perception, e.g., Neely, 1991 for a review.), but planning an utterance—retrieving, ordering, and/or phonologically encoding the lexical items, is especially sensitive to similarity, apparently even when the phonological realization of the referent is a pronoun. It may be conceptual representations that are phonologically realized in the utterance must be kept more active, guiding phonological encoding, than when there is no overt mention in the utterance, and that this longer or stronger activation is a source of higher difficulty. These speculations clearly merit additional research, and they suggest some continued interaction between levels of phonological encoding, where the phonological form is planned, and grammatical encoding, where the sentence form is developed (Janssen and Caramazza, 2009; Jaeger et al., 2012).

Another potentially related literature concerns the use/omission of other optional elements in an utterance, including the richness of inflections attached to a referential form. Kurumada and Jaeger (2013) investigated Japanese speakers’ production of the accusative case marker on direct object nouns such as *student* and *fire engine*; the accusative case marking is optional in spoken Japanese. Kurumada and Jaeger (2013) found higher rates of case marking for sentences that could be more ambiguous for the comprehender, a result that they attributed to producers’ aiming for communicative efficiency, i.e., using case marking when it is more necessary and omitting it when it is less essential. Thus across several different subfields, researchers are examining very closely related phenomena concerning overt mention or omission and addressing questions of choice of form and the forces shaping those choices, so that studies of sentence form and studies of referential form should be able to inform each other.

### ALTERNATIVE ACCOUNTS: MESSAGE FACTORS, AUDIENCE DESIGN, COMMUNICATIVE EFFICIENCY

We interpret speakers’ use of null subjects as emergent from internal interference in speech planning, meaning that at least part of the motivation for omission is driven by producers’ needs. Here we consider some potential alternative interpretations of these results and identify opportunities for future research to shed light on these alternatives.

Because the pictures in picture description experiments necessarily differ across conditions, it is always possible that producers’ utterances are affected by some feature of the pictures other than the target of the experimental manipulation. Thus it is possible that the present null subject findings and Gennari et al. (2012) agentless passive results are due to some differences in the pictures in the semantically similar and dissimilar conditions. For example, the visual salience of to-be-described pictured elements is known to affect speakers’ sentence structures in picture descriptions, perhaps because the task demands may implicitly encourage different amounts of description for visually salient vs. non-salient entities (Montag and MacDonald, 2014). Gennari et al.’s (2012) animate and inanimate entities do differ in salience (Montag and MacDonald, 2014), but in the present study, in which the action picture contains only two entities, both animate and inanimate conditions seem to have highly salient objects. Similarly, in Gennari et al.’s (2012) study with all animate entities, the pictures contained only three salient humans, without any apparent differences in salience across conditions. Thus it seems unlikely that visual salience or other picture properties affected rates of agent omission in the current picture description study or in Gennari et al. (2012) Moreover, there were no pictures in Study 1 here, which found more null subject in the speech/text corpus in all-animate sentences than in ones with inanimate objects.

A second possibility is that the message to be conveyed is different across the different picture conditions in a way that affects the felicity of overt mention of an agent. This possibility seems more relevant to some studies than others. For example, in Gennari et al.’s (2012) all-animate study, the high-similarity participants may have yielded more plausible scenarios than the low-similarity condition. Thus producers may have mentioned the agent of the action (i.e., used full passives like *The builder who was slapped by the astronaut* rather than agentless passives like *the builder who was slapped*) more often in the low similarity condition (astronaut slapping builder) than in the high similarity condition (miner slapping builder) because the low-similarity scene was more unusual, making the astronaut-agent more worthy of mention than the miner-agent. That explanation does not appear to hold for Gennari et al. (2012) animacy manipulations (e.g., holding a vase vs. a baby don’t appear to have wide variations in plausibility) nor does it hold for the Mandarin production study here, where the two conditions were explicitly matched for plausibility. Thus while messages by necessity differ in these animacy/semantic overlap manipulations, they appear not to be an obvious source of variation in pro-drop or other agent omissions.

Another potential alternative interpretation is that the speakers may vary the inclusion/omission of an agent to facilitate listeners’ comprehension, in a form of audience design. On this view, speakers might omit agents that are similar to patients to help comprehenders avoid similarity-based interference. Similarity-based interference does exist in comprehension of at least complex sentences (Acheson and MacDonald, 2011; Van Dyke et al., 2014), but there are also priming effects (facilitation) from semantic overlap in comprehension (see Ledoux et al., 2006, for review). Thus there is not a straightforward argument for how agent omission would help the comprehender under some conditions and not others, and even if there were such an explanation, it is not clear how producers would calculate during online production when an omission would/wouldn’t be helpful to the comprehender. Relatedly, the referential form literature (full noun phrases vs. pronouns) has considered the degree to which choice of form is made for the comprehender (e.g., Arnold et al., 2000; Fukumura et al., 2011). Comprehension studies that compare readers’ processing of repeated full noun phrases vs. pronouns suggest that repeated noun phrases hinder comprehension compared to pronouns (Gordon et al., 1993; Gordon and Hendrick, 1997; Kennison and Gordon, 1997). One study of overt vs. null referential forms in comprehension found that for Mandarin comprehenders, overt pronouns and null forms were both easier than full repeated noun phrases (Yang et al., 1999). Yang et al. (1999) argued that overt and null pronoun forms contributed equally to discourse coherence. This finding does not support an audience design account of the null subject phenomena investigated here. It is likely that in some languages or some situations, the discourse status of null and overt pronouns are different to the point that one form is far more appropriate to convey a producer’s message than another; indeed we saw almost no pronouns in the corpus analysis. In the picture description study, however, Mandarin speakers routinely produced both overt pronouns and null subjects, and their subject omissions are consistent with an explanation based on interference within utterance planning rather than being an audience design strategy to enhance comprehensibility for the perceiver.

In sum, in this as in all examples of variation in utterance form, producers’ choices are likely to be multiply determined by message, production difficulty, and the need to be understood. It is unlikely that a single explanation for a choice of utterance form exists. Indeed, Jaeger et al. (2012) appear to advocate this multi-factor position when they argue that producers’ choices can be traced to communicatively efficent production (Jaeger, 2013; Kurumada and Jaeger, 2013). On this view, choices of inclusion/omission of agents in the present studies and in Gennari et al. (2012) might be viewed as owing to communicative efficiency, that in some cases it is more efficient to omit the agent and in others to include it. Our argument here is not against communicative efficiency or other arguments for multiple forces shaping utterance form. Rather, our position is that “efficiency” needs to be engaged at a more mechanistic level with more specific hypotheses concerning (among other forces) the sources of production difficulty (MacDonald, 2013b). We see the current attempts to link similarity-based interference and choice of utterance form as steps in that direction.

## Conflict of Interest Statement

The authors declare that the research was conducted in the absence of any commercial or financial relationships that could be construed as a potential conflict of interest.

## Footnotes

1 www.pixton.com

2 www.qualtrics.com
